# Brain Plasticity and the Labbé Procedure: Where do we stand?

**DOI:** 10.1016/j.jpra.2025.03.025

**Published:** 2025-04-02

**Authors:** Zhen Yu Wong, Jeremias Schmidt, Frank W. de Jongh, Koen J.A.O. Ingels, Niels van Heerbeek, Sjaak Pouwels

**Affiliations:** a Department of General Surgery, Nottingham City Hospital, Nottingham, United Kingdom; b Department of Plastic, Reconstructive and Aesthetic Surgery, Helios Klinikum Berlin-Buch, Berlin, Germany; c Department of Otorhinolaryngology and Head & Neck Surgery**,** Radboudumc, Nijmegen, The Netherlands; d Department of Surgery, Marien Hospital Herne, University Hospital of Ruhr University Bochum, Herne, NRW, Germany; e Department of Intensive Care Medicine, Elisabeth-Tweesteden Hospital Tilburg, The Netherlands

**Keywords:** Labbé procedure, Facial reanimation surgery, Facial palsy, Peripheral facial palsy, Brain plasticity, laterality

## Abstract

The Labbé procedure is a recognised technique for facial reanimation in patients with facial paralysis (FP). Although it was initially believed that brain plasticity plays a crucial role in the success of this procedure, the evidence supporting this mechanism is limited. This review provides an overview of brain plasticity and its potential application in the Labbé procedure. Additionally, the possibility of trigeminal-facial neurotisation as an alternative mechanism is discussed. Further research is needed to explore the mechanisms to improve the treatment options and enhance early rehabilitation for individuals with FP.

## Introduction

The Labbé procedure, developed by Mr. Daniel Labbé in 1997, is widely acknowledged as an effective technique for facial reanimation in cases of facial paralysis (FP).^1^ Although the procedure has been hypothesised to rely on brain plasticity to facilitate temporalis muscle transition from chewing to smiling, the supporting evidence for this mechanism remains limited. This review aimed to provide a comprehensive overview of brain plasticity and its potential role in the Labbé procedure. This article highlights a niche area within the specialties of plastic facial surgery and neurology. Additionally, it explores alternative mechanisms that may contribute to the procedure's effectiveness.

## The Labbé Procedure

FP is defined as the inability to contract the facial muscles on one or both sides of the face, mainly resulting from temporary or permanent damage to the facial nerve. FP is a debilitating condition that can significantly affect an individual's quality of life. Its causes may vary, including trauma, infection, or nerve damage. The treatment depends on the type of sequelae and can be managed medically through botulinum toxin injections to address synkinesis or surgically to treat the persistent loss of facial nerve function. The surgical approach to the lower face aims to provide movement to the lower part of the face, restore facial symmetry and reproduce a spontaneous natural smile. One of the treatment options available for the chronic sequelae of FP is the Labbé procedure, which is an interesting approach to an infrequently used muscle transfer for FP. The Labbé procedure is a single-step surgical procedure in which the temporalis muscle is lengthened by liberating its coronoid tendon and inserting it into the nasolabial fold.^2^ The advantage of this technique is that it omits the requirement for an intermediate graft and can be performed in a single setting. The temporalis muscle is entirely mobilised towards a new effector, which in this case is the labial commissure. The muscle loses its chewing function, and after 6 months of specific rehabilitation it acquires its new smiling function. Currently, the Labbé procedure remains a second-line surgical option for patients who either decline free muscle transfer or are not suitable for free microvascular muscle transfer, while being recommended to undergo masseter nerve transfer as an adjunct for this procedure.^3^ Nevertheless, the comparative analysis between the Labbé procedure and other facial reanimation surgeries remains limited.^4^

## Brain plasticity

Brain plasticity, also known as neuroplasticity or neural plasticity, refers to the remarkable capacity of the nervous system to undergo adaptive structural and functional changes in response to intrinsic or extrinsic stimuli. This process involves the reorganisation of the brain's structure, functions, or connections, particularly following injuries such as stroke or traumatic brain injury. Neuroplasticity can manifest as beneficial changes that restore lost function, have neutral effects with no significant alterations, or result in negative consequences. It encompasses two major mechanisms: neuronal regeneration or collateral sprouting and functional reorganisation.^5^ Neuronal regeneration involves synaptic plasticity, which allows long-lasting changes in the strength of neuronal connections. It can be influenced by factors such as exercise, environmental stimulation and repetition of tasks, motivation, neuromodulators and medications. Another aspect of neuronal regeneration is adult neurogenesis, the process of generating new neurons in the adult brain. Although adult neurogenesis has been demonstrated in animals, its occurrence and significance in humans are debated. Functional reorganisation involves concepts such as equipotentiality and vicariation, which describe how the brain can redistribute functions to compensate for damage and diaschisis, where damage in one part of the brain leads to functional impairment in connected areas, which further illustrates the interplay of brain regions.

One of the most striking examples of cerebral cortical reorganisation can be observed in patients with brachial plexus injuries who undergo nerve transfers.^6^ In such cases, the intercostal nerves are attached to the musculocutaneous nerve to reconstruct the biceps function. Following reinnervation, patients activate the transfer by taking a deep breath, which leads to elbow flexion. Alfaro et al. demonstrated changes in functional neural patterns, structural connectivity and cortical topography at the visual and auditory cortex in a population using a visual-to-auditory sensory substitution device ‘Eyeborg’ which translates visual information, specifically colours, into corresponding sounds for individuals with colour-blindness.^7^

Understanding brain plasticity is crucial for developing targeted therapies for restoring function and treating symptoms in various neurological conditions. Pharmacological interventions, such as selective serotonin reuptake inhibitors and cholinergic agonists, can also influence brain healing. However, neuroplasticity can have maladaptive aspects, resulting in unwanted symptoms. Different environmental events, such as sensory stimuli, psychoactive drugs, gonadal hormones, parental-child relationships, peer relationships, early stress, intestinal flora, and diet, have been studied for their positive impact on neuroplasticity.^8^ Continued research into the functional connections of the brain will enhance our understanding of neuroplasticity and lead to more effective interventions to facilitate recovery and improve brain health.

## Brain plasticity in the Labbé Procedure

Distinct and consistent patterns of cortical activation can be observed when individuals smile or clench their teeth. These patterns of activation in the cortex indicate that the trigeminal and facial motor cortex areas are separate regions with minimal overlap.^9^ Previous study has shown that cortical representation zones would expand with increased input, to the extent of reshuffling of topographic order while the absence of afferent input would cause neighbouring representational zones to encroach upon the area deprived of its input.^10^ This was further supported by the study from Garmi et al.^11^ Functional magnetic resonance imaging conducted three months post Labbé procedure revealed an increase in the size and intensity of the smile area. Notably, the area associated with smiling tended to extend right up to the area linked to chewing, even merging with it in sagittal view. These findings further support the notion that the motor cortex undergoes reorganisation of the neural pathways involved in facial movement in response to new input. Brain plasticity was further displayed in another study by Blanchin et al., where a facial rehabilitation, ‘mirror-effect’ protocol was used for patients post Labbé procedure.^12^ The mirror-effect protocol involves a patient performing five specific facial movements in front of a monitor equipped with a webcam and an image processing software, which displays a virtual reconstruction of the patient's face with a symmetrical smile. This system provides real-time feedback to the patient, who is required to execute the movements while touching the stimulated area for sensory feedback. The protocol also includes mental imagery exercises and viewing videos of laughter to promote spontaneous smiling through imitation. This ‘positive’ feedback helps to restore the balance between the efferent and afferent pathways, leading to significantly better recovery of facial movement, capitalising on neuroplasticity to promote recovery by exerting stimulus towards the desired cortical areas.

Several hypotheses have been proposed to explain the observed brain plasticity in the Labbé procedure. One hypothesis suggests that new synaptic connections form within the cortex and between the thalamus and cortex.^13^ Another hypothesis suggests that previously inactive inhibitory and excitatory channels become active through GABAergic and glutamatergic pathways.^14^ The utilisation of these circuits by the sensorimotor cortex is believed to play a significant role in the rapid establishment of brain plasticity. These changes in motor area excitability resemble those observed in amputated patients when sensorimotor anaesthetic blocks are used. Another theory was that surviving neurons form new connections with neighbouring muscles with help from neurotrophic and growth factors, guiding the regeneration and reinnervation of neurons.^15^ A recent study pointed out that the lack of original efferent input possibly triggers functional and morphological remodelling of the temporalis muscle through autonomic nerve fibres.^16^

Nevertheless, there is a preexisting overlapping pattern between facial muscle and masseter nerve.^17^ However, the difference in strength of brain plasticity between the Labbé procedure and free muscle transfer innervated by the masseter motor nerve and factors influencing it remain to be investigated.

## Possible alternative mechanisms

Another possible theory proposed by the team is that brain plasticity is not solely responsible for restoring smile in patients with FB. Trigeminal-facial neurotisation of the temporalis muscle, was theorised to be one of the contributing factors for temporalis to lose its chewing function and acquire smiling function.^18^ A study by Petropolus et al. investigated facial muscle reinnervation through the trigeminal pathway following facial nerve paralysis.^19^ Using an animal model, the temporalis transposition procedure was performed at varying time intervals after facial nerve transection. The presence of horseradish peroxidase in the trigeminal nucleus indicated successful trigeminal-facial neurotisation in animals that underwent temporalis transposition within two months of facial denervation. However, this topic remains poorly investigated and inconclusive.

## Conclusion

In conclusion, the Labbé procedure has emerged as a well-established technique for facial reanimation in patients with FP. Initially, it was believed that the success of this procedure relied heavily on brain plasticity, enabling the temporalis muscle to transition from chewing to smiling function. However, the evidence supporting this mechanism remains limited. This review provides insights into the concept of brain plasticity and its potential role in the Labbé procedure. Additionally, it explored the possibility of trigeminal-facial neurotisation as an alternative explanatory mechanism. Further research is warranted to comprehensively compare the Labbé procedure with other facial reanimation surgeries and gain a deeper understanding of the underlying mechanisms. Enhancing our knowledge on facial reanimation will contribute to improved treatment options and effective early rehabilitation strategies for individuals affected by FP.

## Author contributions

**Initial idea:** SP. **Literature search:** ZW, SP. **Writing the article:** ZW, JS, FdJ, KI, NvH, and SP. **Final approval:** ZW, JS, FdJ, KI, NvH, and SP.

## Funding

None.

## Ethical approval

For this type of study formal consent is not required.

## Conflict of interest

The authors have no conflict of interest to report.
